# Supplementary material for: Modeling glioblastoma heterogeneity as a dynamic network of cell states
Source: Mol Syst Biol. 2021 Sep 16;17(9):e10105. doi: 10.15252/msb.202010105 (PMC8444284; doi:10.15252/msb.202010105)
Supplement: Supplementary file 6 — Source Data for Figure 5 [file MSB-17-e10105-s004.zip › Figure5A_sourcedata/GSEA_3071/hallmarks_stateI.GseaPreranked.1623742531165/HALLMARK_INTERFERON_ALPHA_RESPONSE.html]

Details for gene set HALLMARK\_INTERFERON\_ALPHA\_RESPONSE[GSEA]

|  || Dataset | state13071 |
| Phenotype | NoPhenotypeAvailable |
| Upregulated in class | na\_pos |
| GeneSet | HALLMARK\_INTERFERON\_ALPHA\_RESPONSE |
| Enrichment Score (ES) | 0.6109153 |
| Normalized Enrichment Score (NES) | 2.8808253 |
| Nominal p-value | 0.0 |
| FDR q-value | 0.0 |
| FWER p-Value | 0.0 |
Table: GSEA Results Summary

  

Fig 1: Enrichment plot: HALLMARK\_INTERFERON\_ALPHA\_RESPONSE      
 Profile of the Running ES Score & Positions of GeneSet Members on the Rank Ordered List

  

| PROBE | GENE SYMBOL | GENE\_TITLE | RANK IN GENE LIST | RANK METRIC SCORE | RUNNING ES | CORE ENRICHMENT || 1 | NMI |  |  | 131 | 0.962 | 0.0171 | Yes |
| 2 | SAMD9 |  |  | 172 | 0.867 | 0.0551 | Yes |
| 3 | SAMD9L |  |  | 180 | 0.857 | 0.1019 | Yes |
| 4 | B2M |  |  | 241 | 0.744 | 0.1270 | Yes |
| 5 | IFITM3 |  |  | 268 | 0.714 | 0.1603 | Yes |
| 6 | IRF1 |  |  | 276 | 0.705 | 0.1985 | Yes |
| 7 | LGALS3BP |  |  | 286 | 0.696 | 0.2355 | Yes |
| 8 | ISG15 |  |  | 328 | 0.647 | 0.2606 | Yes |
| 9 | PROCR |  |  | 355 | 0.627 | 0.2889 | Yes |
| 10 | EPSTI1 |  |  | 394 | 0.592 | 0.3117 | Yes |
| 11 | IFI44L |  |  | 422 | 0.574 | 0.3367 | Yes |
| 12 | PSMB9 |  |  | 437 | 0.560 | 0.3645 | Yes |
| 13 | ELF1 |  |  | 439 | 0.558 | 0.3961 | Yes |
| 14 | DDX60 |  |  | 481 | 0.527 | 0.4143 | Yes |
| 15 | IFI35 |  |  | 574 | 0.467 | 0.4144 | Yes |
| 16 | IL15 |  |  | 635 | 0.439 | 0.4222 | Yes |
| 17 | PSMB8 |  |  | 669 | 0.423 | 0.4368 | Yes |
| 18 | GBP2 |  |  | 674 | 0.422 | 0.4597 | Yes |
| 19 | BST2 |  |  | 677 | 0.421 | 0.4832 | Yes |
| 20 | ADAR |  |  | 709 | 0.408 | 0.4975 | Yes |
| 21 | IFI44 |  |  | 753 | 0.396 | 0.5077 | Yes |
| 22 | NCOA7 |  |  | 755 | 0.396 | 0.5300 | Yes |
| 23 | IFIT3 |  |  | 767 | 0.393 | 0.5492 | Yes |
| 24 | IFITM2 |  |  | 836 | 0.370 | 0.5507 | Yes |
| 25 | PSMA3 |  |  | 866 | 0.361 | 0.5629 | Yes |
| 26 | IFIH1 |  |  | 962 | 0.336 | 0.5548 | Yes |
| 27 | STAT2 |  |  | 976 | 0.333 | 0.5700 | Yes |
| 28 | CASP1 |  |  | 981 | 0.331 | 0.5877 | Yes |
| 29 | PARP14 |  |  | 1026 | 0.323 | 0.5935 | Yes |
| 30 | LY6E |  |  | 1030 | 0.321 | 0.6109 | Yes |
| 31 | MX1 |  |  | 1130 | 0.302 | 0.5996 | No |
| 32 | PARP12 |  |  | 1244 | 0.279 | 0.5830 | No |
| 33 | UBE2L6 |  |  | 1370 | 0.256 | 0.5617 | No |
| 34 | NUB1 |  |  | 1385 | 0.254 | 0.5721 | No |
| 35 | CNP |  |  | 1582 | -0.265 | 0.5309 | No |
| 36 | GMPR |  |  | 2516 | -0.417 | 0.2862 | No |
Table: GSEA details [plain text format]

  

Fig 2: HALLMARK\_INTERFERON\_ALPHA\_RESPONSE: Random ES distribution      
 Gene set null distribution of ES for **HALLMARK\_INTERFERON\_ALPHA\_RESPONSE**
